# ‘Nasal flossing’: A case report of nasopharyngeal stenosis due to severe erosive lichen planus and a novel therapeutic intervention

**DOI:** 10.1016/j.ijscr.2018.11.002

**Published:** 2018-11-13

**Authors:** Alastair Henry, John Biddlestone, James McCaul

**Affiliations:** a Welsh Deanery, United Kingdom; b University of Glasgow, Speciality Registrar, Scottish Deanery, United Kingdom; c NHS GGC, Professor of Maxillofacial Surgery, University of Bradford, United Kingdom

**Keywords:** OLP, oral lichen planus, NPS, nasopharyngeal stenosis, Oral Lichen planus, Erosive, Nasopharyngeal stenosis, Case report, Nasal flossing

## Abstract

**Introduction:**

We describe a case of severe erosive oral lichen planus that led to nasopharyngeal stenosis. This is a rare clinical presentation that was ultimately, successfully treated by surgery combined with post-operative ‘nasal flossing’: a novel therapeutic intervention.

**Presentation of case:**

A 76-year-old male suffering from a rare case of severe oral lichen planus that was resistant to conservative measures is described. Initial surgery was complicated by recurrence of nasopharyngeal stenosis. Definitive surgery required revision of nasopharyngeal stenosis release combined with a course of post- operative ‘nasal flossing’. The technique for ‘nasal flossing’ is described and demonstrated in photographs. The patient remained asymptomatic at 3 years using this combined approach, with restoration of olfaction, taste perception and voice quality, significantly enhancing quality of life.

**Discussion:**

Erosive oral lichen planus is a rare but important presentation in oral medicine. We found ‘nasal flossing’ to be a successful treatment to maintain nasopharyngeal patency following surgical repair of this uncommon condition. We are not aware that this combined approach has previously been described in the published literature.

**Conclusions:**

Severe erosive oral lichen planus can lead to nasopharyngeal stenosis. Nasopharyngeal stenosis in these patients may be refractive to conventional surgical approaches. ‘Nasal flossing’ is demonstrated to be both practical and acceptable as a surgical adjunct in these difficult to treat cases of recurrent nasopharyngeal stenosis. This report has relevance for all those practicing oral and maxillofacial surgery, ear nose and throat surgery and oral medicine.

## Highlights

• An unusual case of nasopharyngeal stenosis following severe erosive oral lichen planus is described.
• Initial surgical management was complicated by recurrent nasopharyngeal stenosis due to the intense underlying inflammatory disease process.
• ‘Nasal flossing’ is described as a novel technique that can be used as an adjunct to prevent recurrence of nasopharyngeal stenosis and has relevance to a broad range of oral and nose and throat physicians.

## 1 Introduction

Oral Lichen Planus (OLP) is a common chronic inflammatory disease associated with

cell-mediated immunological dysfunction [1]. The prevalence of OLP has been reported as 1.27%. It is characterized by a T-cell mediated response against epithelial basal

cells, leading to basal cell degeneration and sub-epithelial band like infiltration by T- lymphocytes [3]. The aetiology of OLP is not well understood and this is a major obstacle to the development of new therapeutics [4]. Suggested predisposing factors include genetic factors, stress, trauma, and infection [5,6].

There are six different clinical subtypes; reticular, papular, plaque-like, atrophic (erythematous), erosive (ulcerative) and bullous [7]. The most common form is the reticular lesion, which is often asymptomatic. The erosive (ulcerative) type is the second most common and can cause symptoms ranging from a burning sensation to severe pain [8].

The most frequently cited complication of OLP is malignant transformation and 1.1% of patients with OLP develop oral squamous cell carcinoma [3]. Lichen planus at other sites may cause complications due to scarring. Examples include oesophageal stenosis and dysphagia [9], conductive hearing loss in the setting of otic lichen planus [10] and vulval scaring and vaginal stenosis [11].

To our knowledge, there have been no reported cases of nasopharyngeal stenosis (NPS) occurring as a complication of OLP. NPS is an obliteration of the normal communication between the nasopharynx and the oropharynx resulting from the fusion of the tonsillar pillars and soft palate to the posterior pharyngeal wall [12]. NPS is normally managed surgically.

We present a case of NPS caused by OLP, and we also describe a novel technique for prevention of its recurrence following surgical intervention. This work is reported in line with SCARE criteria [13]. The patient was managed in the private practice.

## 2 Presentation of case

**Fig. 1:**
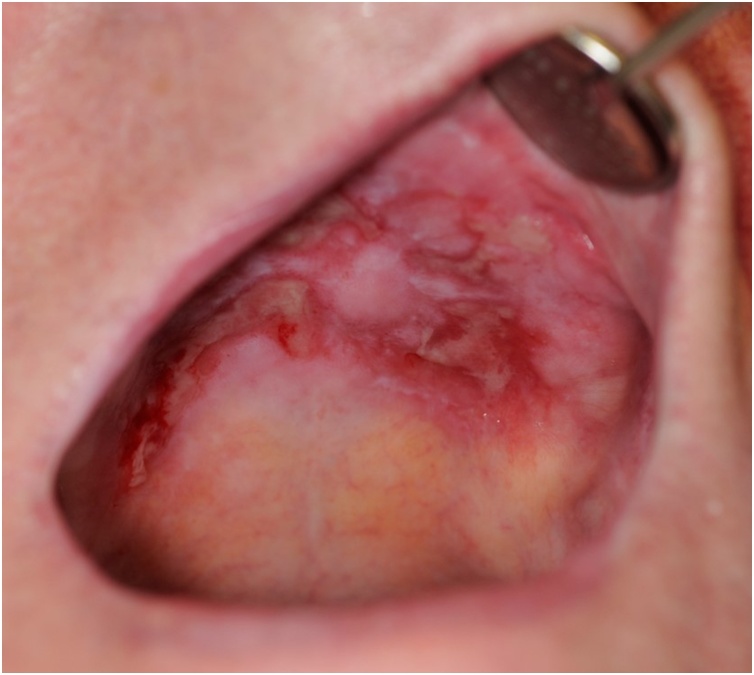
Photographic representation of erosive oral lichen planus at presentation. Significant oral mucosal ulceration is demonstrated affecting both the hard and soft palate.

A 76-year-old retired Caucasian male was referred for an oral and maxillofacial surgery opinion by his medical practitioner complaining of severe oral discomfort, altered phonation and impaired olfaction and gustation. Clinical examination indicated severe erosive lichen planus affecting most surfaces of the oropharynx (Fig. 1). Endoscopic examination also revealed significant involvement of the nasopharynx. The soft palate was particularly involved and had become adhered to the to the posterior pharyngeal wall as a result of the formation of dense scar tissue. This resulted in obstruction of the posterior nasal airway to cause NPS.

Incisional biopsy of a representative area was obtained to confirm the clinical diagnosis. An initial three-month period of medical therapy included fluticasone propionate aqueous nasal spray (50 μg three times daily) and betamethasone oral rinse (500 μg betamethasone tablets dissolved in 20 mL water and used as an oral rinse four times daily).

Following three months of topical corticosteroid use the patient reported compete resolution of oral discomfort but still experienced impaired olfaction and gustation and still perceived changes to voice pitch and resonance.

**Fig. 2:**
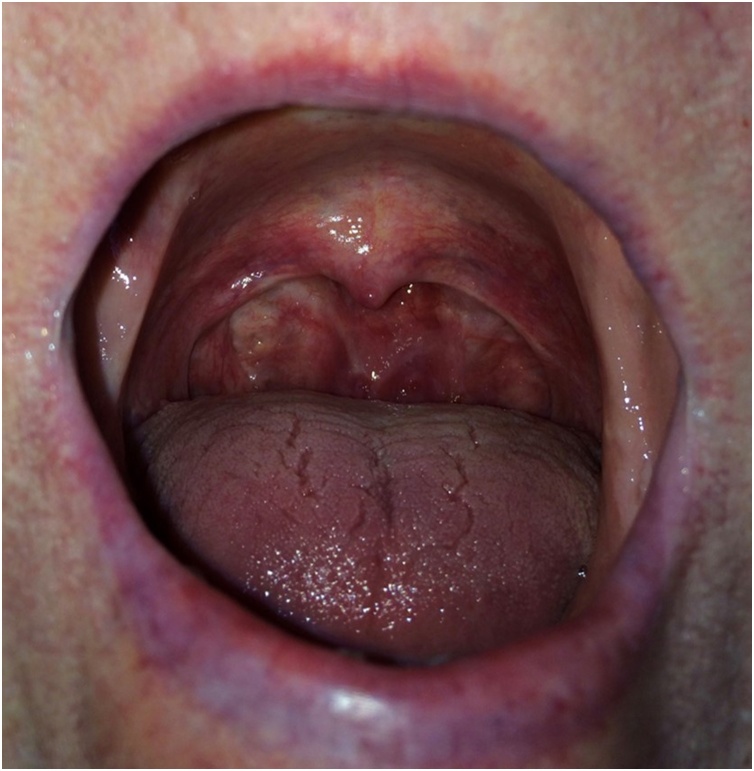
Photographic representation of response to medical therapy. The oral ulceration has largely resolved but the patient still complains symptomatically of nasopharyngeal stenosis.

On examination, the oral mucosa appeared macroscopically healthy (Fig. 2) but the soft palate remained firmly adherent to the posterior pharyngeal wall and the nasal airway remained almost entirely occluded. A further three months of medical therapy resulted in no perceptible improvement in function. Following discussion, the patient opted to undergo surgical division of the nasopharyngeal stenosis under general anaesthetic. Per-oral sharp and blunt dissection was utilised to release the adherent soft palate from the posterior pharyngeal wall. This resulted in a non-epithelialised surface of both the postero-superior soft palate and adjacent nasopharyngeal wall. Complete separation was confirmed intraoperatively using fibre-optic nasoendoscopy. Two silicone nasopharyngeal airways were placed and used as a stent to maintain separation of the soft palate from the pharyngeal wall. These were left in-situ for a period of ten days to allow epithelialisation. The previously described topical corticosteroid regimen was continued.

Four months following the above procedure the patient reported improved olfaction and gustation, and improved tone of voice. Clinical examination confirmed that there was no recurrence of the previously observed NPS.

However, by six months a relapse of erosive OLP had resulted in the recurrence of adhesion between the soft palate and posterior pharyngeal wall. This resulted in NPS and a return of symptoms. The relapse of NPS occurred despite continued use of the topical corticosteroid regimen.

Following informed consent, the patient underwent a second surgical division as described above. Nasopharyngeal airways were used as a stent to allow epithelialisation. Following removal of the nasopharyngeal airways, a novel transnasal-transoral ‘flossing technique’ was employed to maintain separation of the inflamed tissues.

### 2.1 ‘Nasal flossing’ technique

**Fig. 3:**
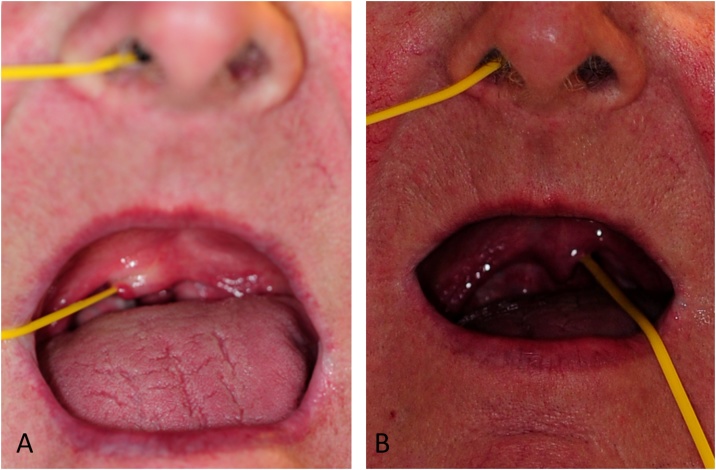
Photographs to depict the technique of ‘nasal flossing’: The silastic sling is introduced through the anterior nares, along the nasal floor and over the superior border of the soft palate before being pulled out through the mouth. This sling allowed gentle traction to be applied to the soft palate so that it was pulled away from the inflamed posterior pharyngeal wall and could also be manoeuvred medially and laterally to free up any early adhesions that had formed. A – Insertion of the sling. B – Lateral movement of the sling.

A 3.5 by 450 mm oval silicone sling (Medasil (Surgical) Ltd, Leeds) was introduced through the anterior nares and advanced along the floor of the nasal cavity until it emerged into the oropharynx so that it could be retrieved and pulled out through the mouth. Gentle traction was applied to both ends of the sling so that the soft palate

was pulled away from the posterior pharyngeal wall. The sling was manoeuvred medially and laterally by angling the oral part of the sling. The procedure was then repeated on the opposite side (Fig. 3A–B).

At outpatient review, two-weeks following surgery, the patient was taught how to insert the sling until it reached the nasopharynx, and then to retrieve the free end by flexing the neck so that the head was in face down position. He then used an explosive cough to expel the free end into the oral cavity where he could retrieve it and manoeuvre it as described above.

There was no recurrence of NPS at one year following the second surgical release. The patient reported normal olfaction, gustation and speech. Nasal flossing was performed daily for a duration of approximately one minute each day. It was well tolerated by the patient, and there were no observed complications during follow-up and preparation of this report. No further surgical intervention has been required at three years

## 3 Discussion

Historically, the main cause of NPS was infection, with the majority of cases being due to tertiary oropharyngeal syphilis [14]. In contemporary practice, NPS is most often due to complications following nasopharyngeal surgery including palatopharyngoplasty, tonsillectomy, adenoidectomy and pharyngeal flap surgery for velopharyngeal insufficiency [14,15]. NPS is also increasingly recognised as a late complication of external beam radiation for head and neck malignancy [16]. Rarely, NPS may be caused by sarcoidosis or cicatricial pemphigoid [17]. There are no previously reported cases NPS as complication of OLP.

Patients with NPS frequently suffer significant morbidities, including phonatory changes, sleep disordered breathing, and otologic disturbances [16].

Steroid therapy is the mainstay of treatment of OLP [18]. A Cochrane review in 2011 found that there were no randomised clinical trials that compared steroids with placebo, and concluded that there is no evidence that one steroid is any more effective than another [19].

The treatment of NPS is challenging as there is a high rate of recurrence [20]. Treatment options vary depending on the location, extent and severity of stenosis. Non-surgical options include local steroid injection, topical mitomycin application, and nasopharyngeal obturator placement [21]. However, most studies support that surgery offers the only option for curative treatment for NPS [22]. Principles include excision of the scar tissue with provision of an epithelial lining [23]. Reported modalities include cold knife excision and laser excision. Skin grafts, local flaps, palatal eversion, regional flaps and microvascular free flaps have all been utilized to provide an epithelial lining [17,20]. Local pharyngeal and soft palatal flaps are the most quoted flaps in literature utilized in the management of NPS [23]. A stent or an obturator may be used to allow epithelisation to occur [23].

In this case, despite an initially successful result achieved by surgical release and stenting, restenosis occurred due to the inherent tendency for the inflamed tissue to reattach.

The multitude of both local and regional methods described in the literature for surgical correction of NPS rely on the availability of healthy tissue in proximity to the site of repair. This was not available in this case due to extensive oro-nasopharyngeal involvement.

In this case the primary repair involved separation of the soft palate from the posterior pharyngeal wall, with minimal excision of scar tissue. Nasopharyngeal airways were then used as stents to maintain position of the soft palate while epithelial migration occurred.

The use of silicone slings and the ‘nasal flossing’ technique described above is a novel approach to maintaining patency of the repair in selected patients. ‘Nasal flossing’ was successful in this case despite the increased tendency for restenosis. The choice of silastic slings (smooth surface) and a gentle technique avoided the effect of the Koebner phenomenon, which is where new lesions occur at sites of trauma.

## 4 Conclusion

We have described a case of severe erosive oral lichen planus where treatment required surgical release and ‘nasal flossing’ to maintain a patent nasopharyngeal airway. To our knowledge, this is the first case of oral lichen planus causing nasopharyngeal stenosis to be reported. This is also the first report of ‘nasal flossing’ as a treatment for this condition.

‘Nasal flossing’ has potential application as an adjunct in the management of any of these causes for nasopharyngeal stenosis, and as such this report has relevance for all those practicing oral and maxillofacial surgery, ear nose and throat surgery and oral medicine.

Use of ‘nasal flossing’ may allow for the utilisation of less complex primary surgical interventions in cases of nasopharyngeal stenosis, as clinicians may have confidence that the patency of the repair can be maintained in the longer term in suitable patients.

## Conflicts of interest

None.

## Funding

This research did not receive any specific grant from funding agencies in the public, commercial, or not-for-profit sectors.

## Ethical approval

This study is a case report and does not require ethical approval in its current form.

## Consent

Written informed consent was obtained from the patient for publication of this case report and accompanying images. A copy of the written consent is available for review by the Editor-in-Chief of this journal on request.

## Authors contribution

AH analysed and interpreted data, drafted the article and approved final version. JB analysed and interpreted data, revised drafted article and approved final version. JM conceived study and acquired data; critically reviewed article and approved final version.

## Registration of research studies

This case report does not require registration as a research study

## Guarantor

The Guarantor is Professor James McCaul.

## Provenance and peer review

Not commissioned, externally peer reviewed.
